# The association between appendicitis severity and patient age with appendiceal neoplasm histology—a population-based study

**DOI:** 10.1007/s00384-022-04132-8

**Published:** 2022-04-26

**Authors:** Jenny Alajääski, Elina Lietzén, Juha M. Grönroos, Jukka-Pekka Mecklin, Ari Leppäniemi, Pia Nordström, Tero Rautio, Tuomo Rantanen, Juhani Sand, Hannu Paajanen, Helena Ollila, Paulina Salminen

**Affiliations:** 1 Department of Surgery, Satasairaala Central Hospital, Pori, Finland; 2 University of Turku, Turku, Finland; 3 Division of Digestive Surgery and Urology, Department of Digestive Surgery, Turku University Hospital, Turku, Finland; 4 Department of Surgery, Nova Hospital, Jyväskylä, Finland; 5 Sport and Health Sciences, Jyväskylä University, Jyväskylä, Finland; 6 Abdominal Center, Helsinki University Hospital, Helsinki, Finland; 7 University of Helsinki, Helsinki, Finland; 8 Division of Surgery, Gastroenterology and Oncology, Tampere University Hospital, Tampere, Finland; 9 University of Tampere, Tampere, Finland; 10 Department of Surgery, Division of Gastroenterology, Oulu University Hospital, Oulu, Finland; 11 University of Oulu, Oulu, Finland; 12 Department of Surgery, Seinäjoki Central Hospital, Seinäjoki, Finland; 13 Department of Surgery, Kuopio University Hospital, Kuopio, Finland; 14 University of Kuopio, Kuopio, Finland; 15 Department of Administration, Tampere University Hospital, Tampere, Finland; 16 Department of Surgery, Mikkeli Central Hospital, Mikkeli, Finland; 17 Clinical Research Center, Turku University Hospital, Turku, Finland

**Keywords:** Appendicitis severity, Appendiceal adenocarcinoma, Neuroendocrine tumor, Pseudomyxoma peritonei, Mixed adeno-neuroendocrine carcinoma, Goblet cell carcinoma

## Abstract

**Purpose:**

Recent studies have reported alarming appendiceal tumor rates associated with complicated acute appendicitis, especially in patients presenting with a periappendicular abscess. However, the data on histology of appendiceal tumors among acute appendicitis patients is limited, especially in patient cohorts differentiating between uncomplicated and complicated acute appendicitis. We have previously reported the association of increased appendiceal tumor prevalence with complicated acute appendicitis in this population-based study. The objective of this secondary analysis was to evaluate the association of both appendicitis severity and patient age with appendiceal tumor histology.

**Methods:**

This nationwide population-based registry study (The Finnish Cancer Registry) was conducted from 2007 to 2013. All appendiceal tumors (*n* = 840) and available medical reports (*n* = 504) of these patients at eight study hospitals were previously evaluated, identifying altogether 250 patients with both acute appendicitis and appendiceal tumor.

**Results:**

The severity of acute appendicitis was significantly associated with more malignant tumor histology. The risk of adenocarcinoma or pseudomyxoma was significantly higher among patients with periappendicular abscess (OR 15.05, CI 95% 6.98–32.49, *p* < 0.001) and patients presenting with perforated acute appendicitis (OR 4.09, CI 95% 1.69–9.90, *p* = 0.0018) compared to patients with uncomplicated acute appendicitis. Similarly, patient age over 40 years was significantly associated with the risk of adenocarcinoma and pseudomyxoma (OR 26.46, Cl 95% 7.95–88.09, *p* < 0.001). Patient sex was not associated with a more malignant appendiceal tumor histology (*p* = 0.67).

**Conclusion:**

More malignant appendiceal tumor histology of adenocarcinoma or pseudomyxoma was significantly associated with patient age over 40 years and complicated acute appendicitis, especially periappendicular abscess.

## Introduction

Acute appendicitis occurs in 7–12% of the general population, and appendectomy is one of the most common indications for emergency abdominal surgery [1–3]. Uncomplicated and complicated acute appendicitis seems to follow different epidemiological and clinical patterns, also suggesting potentially different pathophysiology behind these two different forms of appendicitis severity [2, 3]. In computed tomography (CT) confirmed uncomplicated acute appendicitis, antibiotic therapy has been proven to be safe, efficient, and feasible both at short- and long-term follow-up [4–7]. Uncomplicated acute appendicitis may even resolve with only symptomatic treatment [8, 9], and the over century-old treatment paradigm of appendectomy for all may not be necessary for the majority of patients with CT confirmed uncomplicated acute appendicitis [4–9]. Approximately 25 to 35% of acute appendicitis cases present as complicated [3]. Acute appendicitis presenting with gangrene, perforation, periappendicular abscess, or tumor has been traditionally considered complicated acute appendicitis usually requiring emergency appendectomy. In addition, the presence of an appendicolith has been shown to be associated with a more complicated course of acute appendicitis [7, 10]. CT is the gold standard in diagnosing acute appendicitis also able to quite accurately differentiate between uncomplicated and complicated acute appendicitis [11–13]. In 3 to 10% of patients, complicated appendicitis presents as a closed, circumscribed periappendicular abscess [14–16].

Management of periappendicular abscess usually involves the initial nonoperative treatment with antibiotics and drainage (if necessary), followed by interval appendectomy. Previously the rationale for promoting interval appendectomy has been the prevention of recurrent appendicitis, but the reported recurrence risk after initial successful nonoperative management of periappendicular abscess is quite low, varying between 5 and 20% [14–16]. The reported overall risk of an underlying malignant neoplasm in acute appendicitis is very low, varying between 0.7 and 3% [17–21]. However, there are many recent studies reporting an alarming rate of appendiceal neoplasms detected at interval appendectomy in patients with previous periappendicular abscess [22–27]. A recent randomized controlled trial comparing interval appendectomy to follow-up with magnetic resonance imaging in patients with initial successful antibiotic treatment of periappendicular abscess was prematurely terminated based on high tumor rate in the interval appendectomy group with an overall appendiceal neoplasm incidence of 20% (12 of 60), and all of these patients were over 40 years of age [24].

The World Health Organisation (WHO) classifies appendiceal tumors in two main groups: neuroendocrine tumors (NETs) [28, 29] and appendiceal carcinomas (colonic-type and mucinous-type) [30] ranging from NET 5-year survival rate of 100% for a localized and 85–100% for a regional disease [31] to 5-year survival of 40–75% of mixed type NETs (MANECs, mixed adeno-neuroendocrine carcinomas) [30, 32, 33] and goblet cell carcinomas and to the 5-year survival of 48–58% of colonic-type appendiceal adenocarcinomas [34, 35]. The 2010 WHO classification also recognizes three main categories of mucinous neoplasms: mucinous adenoma, low-grade appendiceal mucinous neoplasm (LAMN), and mucinous adenocarcinoma. Although mucinous tumors are considered to be benign, these appendiceal neoplasms can progress to peritoneal dissemination resulting in the condition defined as pseudomyxoma peritonei.

Data on the association of appendicitis severity with appendiceal neoplasm incidence is limited. To our knowledge, there are no population-based registry studies published on the tumor histology association with appendicitis severity. We have previously reported the association of appendiceal neoplasm risk with complicated acute appendicitis in this population-based study [36]. The objective of this secondary analysis was to evaluate the association of appendiceal tumor histology with both the severity of acute appendicitis and patient age and sex.

## Methods

The study design, rationale, and methods for this nationwide population-based registry study have been previously reported [36]. Briefly, all appendiceal tumors in Finland during 2007–2013 were collected from the Finnish Cancer Registry (FCR), maintaining a nationwide database on all cancer cases in Finland. From this patient population of histologically proven appendiceal primary tumors, we collected hospital medical record data on patients treated at eight study hospitals: all five university hospitals (Turku, Helsinki, Tampere, Oulu, and Kuopio) and three larger central hospitals (Jyväskylä, Lahti, and Mikkeli) representing 70% of the whole population in Finland. This secondary analysis focused on evaluating detailed appendiceal tumor histology association with appendicitis severity and patient demographics of age and sex. Patients were divided into three groups based on the appendiceal neoplasm histology: group 1 NETs, group 2 MANECs and goblet cell carcinomas, and group 3 adenocarcinomas and pseudomyxomas (including mucinous adenoma, low-grade appendiceal mucinous neoplasm (LAMN), and mucinous adenocarcinoma). Diagnoses were classified according to the WHO International Classification of Disease year 2010 classification (ICD-10). This study was approved by the Turku University Hospital ethical committee.

### Statistical methods

The data were presented as means with standard deviation (SD) for continuous variables (age) and counts with percentage for categorical variables (sex). The differences in background variables between the three groups (uncomplicated acute appendicitis vs perforated acute appendicitis vs periappendicular abscess) were tested for a numeric variable with one-way analysis of variance and for categorical variables using the chi-square test (Tables 1 and 2). Fisher’s exact test was used if the variable had low group frequencies.

**Table 1:** Study population: patient characteristics

	**Uncomplicated acute appendicitis *n* = 148**	**Perforated acute appendicitis *n* = 36**	**Peri-appendicular abscess *n* = 66**	**All patients *n* = 250**	***p***
**Age, years**					< 0.001
Mean SD	41 18	56 18	58 15	47 19	
**Sex** *n* (percentages)					0.005
Men Women	73 (49%) 75 (51%)	17 (47%) 19 (53%)	21 (32%) 45 (68%)	111 (44%) 139 (56%)	
**Preoperative imaging** *n* (percentages)					< 0.001
Contrast enhanced CT ^a^ CT without contrast Ultrasound MRI ^b^ PET ^c^-CT No imaging	58 (39%) - 29 (20%) 4 (3.0%) 2 (1.0%) 55 (37%)	27 (75%) - 3 (8.0%) 1 (3.0%) - 5 (14%)	53 (80%) 1 (2.0%) 8 (12%) - - 4 (6.0%)	138 (55%) 1 (0.4%) 40 (16%) 5 (2.0%) 2 (0.8%) 64 (25%)	
**Surgery** *n* (percentages)					< 0.001
Emergency Elective	142 (96%) 6 (4.0%)	36 (100%) -	37 (56%) 29 (44%)	215 (86%) 35 (14%)	
**Macroscopic tumor suspicion** *n* (percentages)					< 0.001
Yes No Unclear	8 (5.0%) 137 (93%) 3 (2.0%)	1 (3.0%) 34 (94%) 1 (3.0%)	18 (27%) 48 (73%) -	27 (11%) 219 (88%) 4 (1.0%)	
**Primary operation** *n* (percentages)					< 0.001
Open appendectomy Laparoscopic appendectomy Ileocecal resection Right hemicolectomy	103 (70%) 42 (28%) 2 (1.3%) 1 (0.7%)	28 (78%) 5 (14%) 2 (5.0%) 1 (3.0%)	27 (41%) 24 (36%) 8 (12%) 7 (11%)	158 (63%) 71 (28%) 12 (5.0%) 9 (4.0%)	
**Metastasis** *n* (percentages)					< 0.001
No Local Disseminated	134 (91%) 5 (3.0%) 9 (6.0%)	28 (78%) 1 (3.0%) 7 (19%)	38 (58%) 4 (6.0%) 24 (36%)	200 (80%) 10 (4.0%) 40 (16%)	
**Additional operation** *n* (percentages)					0.049
Ileocecal resection Right hemicolectomy HIPEC ^d^ Other None	6 (4.0%) 35 (24%) 2 (1.4%) 1 (0.7%) 104 (70%)	6 (17%) 16 (44%) - - 14 (39%)	3 (4.5%) 32 (48%) 2 (3.0%) 4 (6.0%) 25 (38%)	15 (6.0%) 83 (33%) 4 (1.6%) 5 (2.0%) 143 (57%)	

^a^ *CT*, computed tomography

^b^ *MRI*, magnetic resonance imaging

^c^*PET*, positron emission tomography

^d^ *HIPEC*, hyperthermic intraperitoneal chemotherapy

**Table 2:** The association of appendiceal tumor histology with the severity of acute appendicitis, gender, and age of the patient

	**NETs **^**a**^ ***n* = 142**	**MANECs **^**b**^** and goblet cell tumors *n* = 38**	**Adenocarcinomas and pseudomyxomas** ***n* = 70**	**All patients** ***n* = 250**	***p***
**Severity of acute appendicitis** *n* (percentages)					< 0.001
Uncomplicated appendicitis Complicated appendicitis - Perforation - Periappendicular abscess	110 (74%) 32 (31%) 17 (47%) 15 (23%)	19 (13%) 19 (19%) 7 (19%) 12 (18%)	19 (13%) 51 (50%) 12 (33%) 39 (59%)	148 (59%) 102 (41%) 36 (14%) 66 (26%)	
**Sex** *n* (percentages)					0.670
Male Female	65 (59%) 77 (55%)	18 (16%) 20 (14%)	28 (25%) 42 (30%)	111 (44%) 139 (56%)	
**Age, years**					< 0.001
Mean SD	39 18	55 13	61 12	47 19	
**Age** *n* (percentages)					< 0.001
< **40 years** ** ≥ 40 years**	77 (91%) **65 (39%)**	5 (5.9%) **33 (20%)**	3 (3.5%) **67 (41%)**	85 (34%) **165 (66%)**	

^**a**^ *NETs*, neuroendocrine tumors

^**b**^ *MANECs*, mixed adeno-neuroendocrine carcinomas

The association between appendiceal tumor histology and the severity of acute appendicitis, sex, and age of the patient was examined using multinomial logistic regression. Age was also examined as a categorical variable (under 40 years vs 40 years and older). The explanatory factors were first modeled separately and then together in the same model. The level of significance was set at a *p*-value < 0.05. All of the statistical analyses were performed using SAS version 9.4 (SAS Institute Inc, Cary, NC, USA).

## Results

**Fig. 1:**
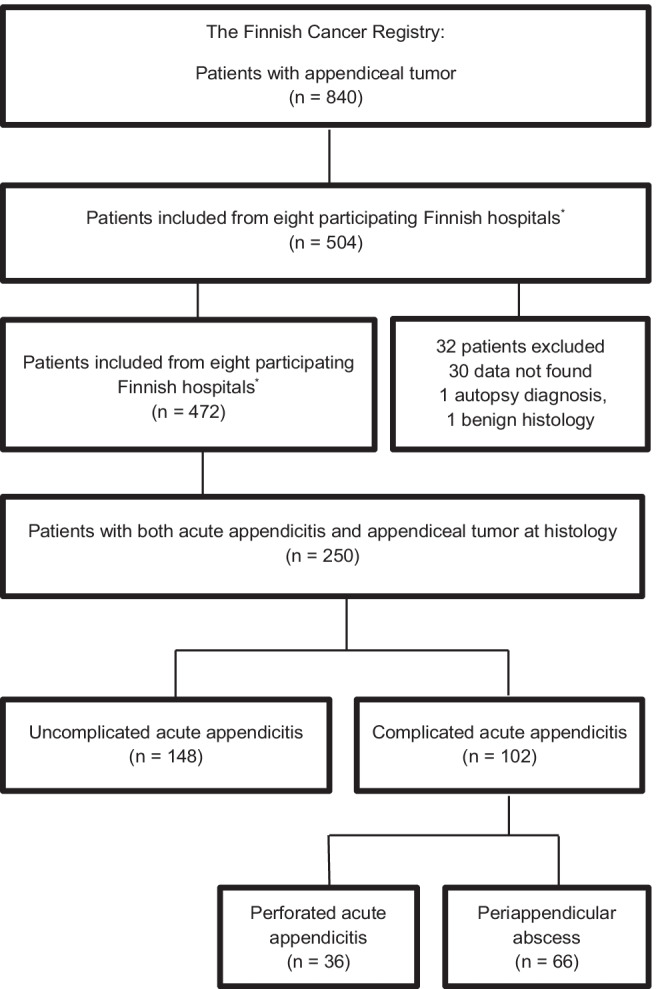
Patient inclusion from The Finnish Cancer Registry (FCR) between years 2007 and 2013

As previously reported [36], there were altogether 250 patients with available diagnostic and clinical data included in this study with both acute appendicitis and appendiceal tumor based on both surgical findings and histology (Fig. 1). Out of these, 148 (59%) had uncomplicated and 102 (41%) complicated acute appendicitis, with the latter group consisting of 36 patients with perforation and 66 patients with a periappendiceal abscess. Detailed patient characteristics of the study population are presented in Table 1. The majority of the patients (86%, 215/250) underwent emergency surgery, with the majority of patients (66%, 142/215) ending up with a diagnosis of uncomplicated acute appendicitis at histopathology in addition to the tumor. The main primary operation was laparoscopic or open appendectomy (91%, 229/250) regardless of the severity of appendicitis. There were altogether 35 elective operations, and 85% (29/35) of these were performed to patients presenting with a periappendicular abscess: 74% (26/35) of all elective operations were interval appendectomies, 11% (4/35) were ileocecal resections, and 14% (5/35) were right hemicolectomies. All hemicolectomies were performed in situations with intraoperative clinical suspicion of an appendiceal neoplasm. During the surgery, clinical tumor suspicion was aroused significantly more often in the case of periappendicular abscess (27%) compared with uncomplicated acute appendicitis (5%) and in patients with perforated (3%) acute appendicitis (*p* < 0.001). In the whole study population, no metastasis was found in 200 (80%) cases, but in case of metastasis, the disease was significantly more often disseminated in the case of periappendicular abscess (24/66, 36%) than in uncomplicated (9/148, 6%) or perforated (7/36, 19%) acute appendicitis (*p* < 0.001).

Out of the 250 patients with both appendiceal tumor and acute appendicitis, 142 patients (57%) had NET, 38 patients (15%) had MANEC or goblet cell carcinoma, and 70 patients (28%) presented with adenocarcinoma or pseudomyxoma (Table 2). Among patients with uncomplicated acute appendicitis (*n* = 110), the incidence of NETs was highest at 74%, and NETs were also the most common tumor in perforated acute appendicitis (17/36, 47%). In patients with periappendicular abscess, the most prevalent tumors (39/66, 59%) were adenocarcinomas and pseudomyxomas. The severity of acute appendicitis was associated with more malignant tumor histology. The risk of adenocarcinoma and pseudomyxoma was significantly higher among both patients with periappendicular abscess (OR 15.05, CI 95% 6.98–32.49, *p* < 0.001) and patients with perforated acute appendicitis (OR 4.09, CI 95% 1.69–9.90, *p* = 0.0018) compared to patients with uncomplicated acute appendicitis. The risk of MANEC and goblet cell carcinoma was higher in patients with periappendicular abscess (OR 4.63, CI 95% 1.88–11.41, *p* = 0.0009) compared with uncomplicated acute appendicitis, but not significantly higher in patients with perforated acute appendicitis (OR 2.38, CI 95% 0.87–6.52, *p* = 0.091).

Patient age was significantly associated with appendiceal neoplasm histology. The risk of adenocarcinoma or peudomyxoma was significantly higher in older patients (OR 5.32, Cl 95% 3.34–8.46, *p* < 0.001). Among patients under the age of 40 years, almost all (91%) appendiceal tumors were NETs. In patients over 40 years, the risk for adenocarcinomas or pseudomyxomas was significantly higher than in patients under 40 years of age (41 vs. 3.5%) (OR 26.46, CI 95% 7.95–88.09, *p* < 0.001). Both the age of the patient and the severity of acute appendicitis were independent risk factors for more malignant tumor histology (*p* = 0.330). Out of the 250 patients, 56% (*n* = 139) were female and 44% (*n* = 111) were male. Patient sex was not associated with the appendiceal tumor histology (*p* = 0.670). The risk for more malignant tumor histology did not differ between women and men; for women, the risk of MANEC/goblet cell carcinoma and adenocarcinoma/pseudomyxoma was not higher compared with men (OR 0.94, CI 95% 0.46–1.92, *p* = 0.860, and OR 1.27, CI 95% 0.71–2.26, *p* = 0.430, respectively).

## Discussion

In this study, both patient age and the severity of acute appendicitis ranging from uncomplicated acute appendicitis to perforated appendicitis and to periappendicular abscess were associated with more malignant histology of the appendiceal neoplasm. The risk of adenocarcinoma or pseudomyxoma was significantly higher among patients over 40 years of age and patients presenting with either periappendicular abscess or perforated acute appendicitis compared with uncomplicated acute appendicitis. Similarly, the higher risk of MANEC and goblet cell carcinoma was associated with periappendicular abscess. Patient sex was not associated with a more malignant appendiceal tumor histology.

The role of interval appendectomy after successful non-operative management of periappendicular abscess is still debated as the reported risk of appendicitis recurrence after the initial successful nonoperative management of periappendicular abscess is quite low [14, 37]. In a small randomized trial comparing emergency laparoscopic appendectomy with antibiotic therapy in the treatment of periappendicular abscess, they reported a 5% prevalence of appendiceal neoplasms [38]. However, other larger studies have reported contradictory alarming rates of appendiceal neoplasms in patients presenting with complicated acute appendicitis and especially associated with periappendicular abscess [23–27]. In this population-based registry study, we have previously reported a significantly higher risk of an appendiceal neoplasm associated with complicated acute appendicitis compared to an uncomplicated form of the disease (3.24 vs. 0.87%), and this risk was even higher in a subgroup analysis comparing periappendicular abscess to uncomplicated acute appendicitis (4.99 vs. 0.87%) [36]. A recent randomized clinical trial by our study group [24] compared interval appendectomy and follow-up with magnetic resonance imaging (MRI) after initial successful non-operative treatment of periappendicular abscess. This trial was prematurely terminated owing to the high rate (17% at interim analysis in the interval appendectomy group with a final rate of 20%) of appendiceal tumors associated with periappendicular abscess. All of the appendiceal tumors were diagnosed in patients over 40 years of age [24]. Similar alarming appendiceal neoplasm rates have been reported in other studies, with appendiceal tumor rates ranging from 10 to 29% [23, 25, 26]. The results of the current secondary analysis of our population-based study corroborate these findings and further support the need for interval appendectomy after initial non-operative management of complicated acute appendicitis. If the high rate of appendiceal neoplasms after periappendicular abscess is validated by future prospective cohorts, appendectomy in the initial acute phase should not be promoted as this would transform a restricted tumor perforation to an unlimited peritoneal spreading.

In addition to appendicitis severity, patient age was significantly associated with more malignant histology of the appendiceal neoplasm. Among patients over 40 years old, the risk of adenocarcinoma or pseudomyxoma was significantly higher compared with patients under 40 years (41 vs. 3.5%), corroborating the findings of previous studies [23–26].

The strong element of this secondary analysis of the nationwide population-based study [36] is the comprehensive analysis of appendiceal tumor risk and histology between uncomplicated and complicated acute appendicitis. To our knowledge, there are no other studies reporting the association of more malignant histology with increasing appendicitis severity from uncomplicated acute appendicitis to periappendicular abscess. A limitation of this study is that there are only eight study hospitals included instead of the whole Finnish Cancer registry. However, these study hospitals include all Finnish university hospitals and largest central hospitals, representing 70% of the Finnish population and 60% of the registry patient population.

## Conclusion

More malignant appendiceal tumor histology of adenocarcinoma or pseudomyxoma was significantly associated with patient age over 40 years and complicated acute appendicitis, especially periappendicular abscess.

## Data Availability

If interested in the study data and individual patient data, please contact the corresponding author and the principal investigator, and data sharing will be evaluated based on the request.
